# Standardized assessment of vascular reconstruction kernels in photon-counting CT angiographies of the leg using a continuous extracorporeal perfusion model

**DOI:** 10.1038/s41598-023-39063-z

**Published:** 2023-07-26

**Authors:** Philipp Gruschwitz, Viktor Hartung, Florian Kleefeldt, Süleyman Ergün, Sven Lichthardt, Henner Huflage, Robin Hendel, Andreas Steven Kunz, Pauline Pannenbecker, Philipp Josef Kuhl, Anne Marie Augustin, Thorsten Alexander Bley, Bernhard Petritsch, Jan-Peter Grunz

**Affiliations:** 1 Department of Diagnostic and Interventional Radiology, University Hospital of Würzburg, Oberdürrbacher Str. 6, 97080 Würzburg, Germany; 2 Institute of Anatomy and Cell Biology, University of Würzburg, Würzburg, Germany; 3 Department of General, Visceral, Transplant, Vascular, and Pediatric Surgery, University Hospital of Würzburg, Würzburg, Germany

**Keywords:** Experimental models of disease, Preclinical research, Translational research

## Abstract

This study evaluated the influence of different vascular reconstruction kernels on the image quality of CT angiographies of the lower extremity runoff using a 1st-generation photon-counting-detector CT (PCD-CT) compared with dose-matched examinations on a 3rd-generation energy-integrating-detector CT (EID-CT). Inducing continuous extracorporeal perfusion in a human cadaveric model, we performed CT angiographies of eight upper leg arterial runoffs with radiation dose-equivalent 120 kVp acquisition protocols (CTDI_vol_ 5 mGy). Reconstructions were executed with different vascular kernels, matching the individual modulation transfer functions between scanners. Signal-to-noise-ratios (SNR) and contrast-to-noise-ratios (CNR) were computed to assess objective image quality. Six radiologists evaluated image quality subjectively using a forced-choice pairwise comparison tool. Interrater agreement was determined by calculating Kendall’s concordance coefficient (*W*). The intraluminal attenuation of PCD-CT images was significantly higher than of EID-CT (414.7 ± 27.3 HU vs. 329.3 ± 24.5 HU; p < 0.001). Using comparable kernels, image noise with PCD-CT was significantly lower than with EID-CT (p ≤ 0.044). Correspondingly, SNR and CNR were approximately twofold higher for PCD-CT (p < 0.001). Increasing the spatial frequency for PCD-CT reconstructions by one level resulted in similar metrics compared to EID-CT (CNR_fat_; EID-CT Bv49: 21.7 ± 3.7 versus PCD-CT Bv60: 21.4 ± 3.5). Overall image quality of PCD-CTA achieved ratings superior to EID-CTA irrespective of the used reconstruction kernels (best: PCD-CT Bv60; worst: EID-CT Bv40; p < 0.001). Interrater agreement was good (*W* = 0.78). Concluding, PCD-CT offers superior intraluminal attenuation, SNR, and CNR compared to EID-CT in angiographies of the upper leg arterial runoff. Combined with improved subjective image quality, PCD-CT facilitates the use of sharper convolution kernels and ultimately bears the potential of improved vascular structure assessability.

## Introduction

Contrast-enhanced computed tomography angiography (CTA) constitutes the first-line diagnostic modality in the evaluation of the peripheral arterial circulation^1,2^. By providing a rapid depiction of the vascular status, CTA primarily aids in evaluating further treatment options and thus potential indications for surgical or interventional therapy to avoid unnecessary or probably unsuccessful procedures^3^. However, CTA bears certain limitations, especially for the depiction of the smaller vasculature, e.g., the arteries in the lower leg and the perforating femoral vessels. Furthermore, vessel lumen assessment is particularly limited in patients with heavy arterial wall calcifications. The introduction of dual-energy CT and associated postprocessing options have not been able to alleviate these drawbacks satisfactorily^4–6^. However, the latest clinical photon-counting detector CT (PCD-CT) technology seems to be able to address these issues. The semiconductor design allows for direct measurement of each photon’s energy, thus revoking the previously required intermediate step of converting the photons to light. This development also eliminates the need for isolating septa within the detector and allows for smaller overall pixel size, thus increasing spatial resolution^7,8^. Furthermore, background noise and beam hardening can be reduced significantly^9^. Independently, the selection of different reconstruction kernels is known to affect resolution and image noise^10,11^. While a variety of postprocessing options have been investigated for cardiac^11–16^, head/neck^17,18^, and aortic PCD-CT^19–23^, suchlike prospectively designed investigations regarding peripheral vessels remain scarce^24^.

To close this scientific gap, this study aimed to investigate the influence of different vascular convolution kernels on the image quality of PCD-CT in comparison to dose-matched energy-integrating detector CT (EID-CT) examinations in a continuous extracorporeal perfusion model.

## Materials and methods

### Cadaveric specimens and extracorporeal perfusion model

The anatomical institute of the local university provided four fresh-frozen body donors which were subsequently included in this experimental study. Donors had written informed consented to the posthumous use for study and research purposes. Experiments were approved by Institutional Review Board of the University of Würzburg (protocol number: 20220413 01), who waived the need for further informed consent, and were conducted according all applicable laws and regulations.

**Figure 1:**
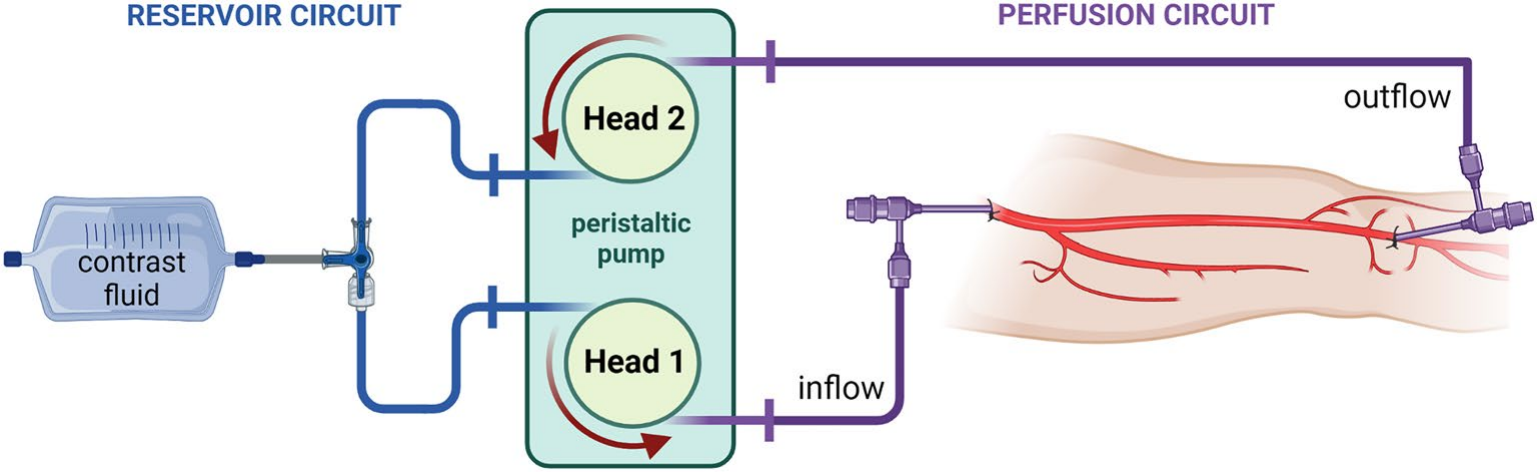
Perfusion circuit schematics. (Created with BioRender.com).

After surgical dissection of the common femoral and popliteal artery, as well as insertion of introduction sheaths, a continuous extracorporeal perfusion was established by means of a peristaltic pump. To this effect, a mixture of Ringer’s solution, glucose solution and iodine-containing contrast medium (Peritrast® 400 mg/ml, Dr. Franz Köhler Chemie GmbH, Bensheim, Germany) with an effective iodine concentration of 12.9 mg/ml was used. A schematic illustration of the experimental setup is provided in Fig. 1. Detailed background information on the establishment of the extracorporeal perfusion model and the necessary materials can be found elsewhere^25^.

### Technical specifications and scan protocols

A clinical 1st-generation PCD-CT (Naeotom Alpha; Siemens Healthcare GmbH, Forchheim, Germany) and a 3rd-generation dual-source EID-CT (Somatom Force, Siemens Healthcare GmbH) were employed to perform CTA of the upper leg runoff. Scans were generated with a fixed tube voltage of 120 kVp and effective tube currents of 63 mAs (PCD-CT) and 74 mAs (EID-CT), respectively, to achieve matching radiation doses (CTDI_Vol_ = 5 mGy). Standard collimations of 144 × 0.4 mm (PCD-CT) and 96 × 0.6 mm (EID-CT) were used with an identical pitch factor of 1.0 and a rotation time of 0.5 s.

### Image reconstruction parameters

Images were reconstructed individually for each leg with a field of view of 150 mm, a 512 × 512 pixel matrix and a slice thickness/increment of 1.0 mm, each. PCD-CT and EID-CT datasets were each reformatted with three vendor-specific vascular kernels characterized by different spatial frequencies (ρ_50_: spatial frequency at the 50% value of the modulation transfer function): For PCD-CT scans, Bv40 (ρ_50_ = 3.95 lp/cm), Bv48 (ρ_50_ = 5.40 lp/cm), and Bv60 (ρ_50_ = 8.79 lp/cm) were employed. Matched to the respective PCD-CT modulation transfer functions, Bv40 (ρ_50_ = 3.95 lp/cm), Bv49 (ρ_50_ = 5.62 lp/cm), and Bv59 (ρ_50_ = 8.32 lp/cm) were selected for EID-CT scans to achieve the best possible comparability. Furthermore, a sharp vascular kernel (Bv76, ρ_50_ = 16.47 lp/cm) only available for PCD-CT data was run. Of note, other relevant spatial frequency values (at 10% and 2% of the modulation transfer function) are included in Supplemental Table S1. Based on vendor information, comparable iterative reconstruction levels were chosen for PCD- and EID-CT datasets. EID-CT raw data was reformatted using a 3rd generation iterative reconstruction algorithm (level 3, ADMIRE, Siemens Healthcare GmbH) and likewise a 4th generation algorithm (level 3, QIR, Siemens) with PCD-CT. It must be noted that the latter algorithm prevents the counting of low-energy photons below a threshold of 20 keV, thereby suppressing electronic background noise.

### Objective image analysis

**Figure 2:**
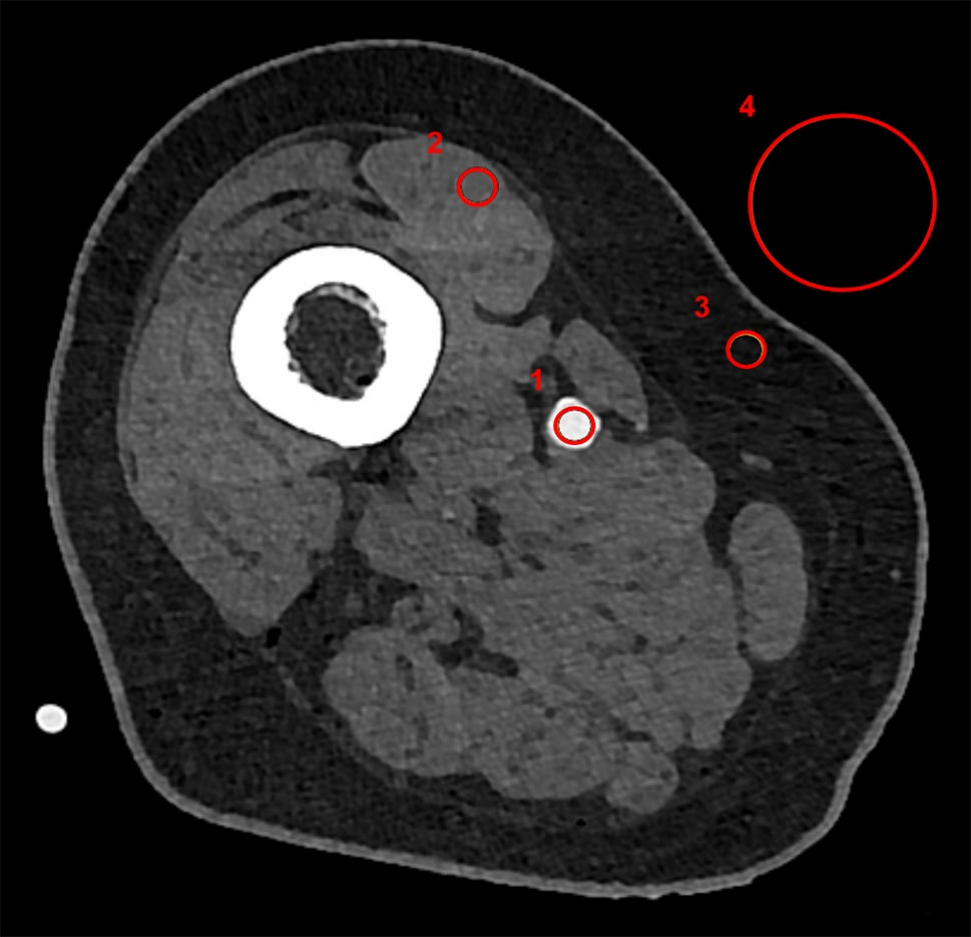
Axial slice of the right upper leg displaying ROI placement. (**1**) ROI 50 mm^2^—HU arterial lumen, absolute HU value and standard deviation. (**2**) ROI 50 mm^2^—HU muscular tissue, absolute HU value. (**3**) ROI 50 mm^2^—HU fat tissue, standard deviation. (**4**) ROI 400 mm^2^—HU air, standard deviation.

Density in Hounsfield Units (HU) within vessel lumina and the surrounding muscle tissue was measured by means of standardized regions of interest (ROI). Each upper leg arterial runoff was evaluated at four consistent levels (proximal, middle, distal superficial femoral artery, and popliteal artery). In order to estimate image noise, the standard deviation of the attenuation (HU) in standardized ROIs within the arteries as well in subcutaneous fat tissue and surrounding air were used. All measurements were performed by one radiologist with 5 years of experience in the field with a clinical picture archiving and communication system (Merlin, Phönix-PACS, Freiburg, Germany) as is presented in Fig. 2. Signal-to-noise ratios (SNR) and contrast-to-noise-ratios (CNR) were computed as *SNR* = *HU*_*artery*_*/SD*_*fat*_ or rather *CNR* = *(HU*_*artery*_* – HU*_*muscle*_*)/SD*_*fat*_.

### Subjective image analysis

Subjective image quality of the reconstructed image stacks generated was compared using a browser-based forced-choice pairwise comparison tool. Representative image slices of both legs at the proximal and distal levels were presented in random order (7 kernels × 2 legs × 2 levels × 4 donors = 112 images), resulting in 336 side-by-side comparisons (21 comparisons for 16 slice positions). Assessment was performed independently by six radiologists with 2 to 8 years of experience in CTA imaging without imposing a time limit or providing technical information. Raters judged overall image quality and luminal assessability using a certified diagnostic monitor (RadiForce RX660; EIZO, Hakusan, Japan). It may be noted that the pairwise comparison tool was chosen over a conventional rating-based analysis in order to simplify the assessment of images that show mostly minor differences. Furthermore, the setup allows for a significant reduction of individual reader effort and time requirement.

### Data analysis and statistics

Generated data were processed in a Jupyter Notebook environment using Python version 3.8.15 and additional freely available libraries (pandas 1.3.5, numpy 1.21.6, choix 0.3.5, kendall w 1.0.0). Dedicated software (DATAtab e.U., Graz, Austria) was utilized for statistical analysis. Continuous variables are given as mean ± standard deviation. Significance is indicated by an alpha level of p < 0.05. Objective image parameters were compared using paired t-test (pair-by-pair) or one-way analyses of variance (ANOVA) and Bonferroni-corrected pairwise post-hoc tests (group). For comparison of the subjective image quality ratings, Friedman tests were employed. By fitting a Bradley-Terry-Model in the choix library^26^, preference ranks were calculated for the entire dataset and for each rater individually, resulting in a final hierarchic ranking of the compared subgroups from 1 (best) to 7 (worst). Bump chart diagrams and Boxplots were computed to visualize the results of objective and subjective image quality analyses. Inter-reader agreement was computed by calculation of Kendall’s concordance coefficient (*W*).

## Results

### Objective image analysis

**Figure 3:**
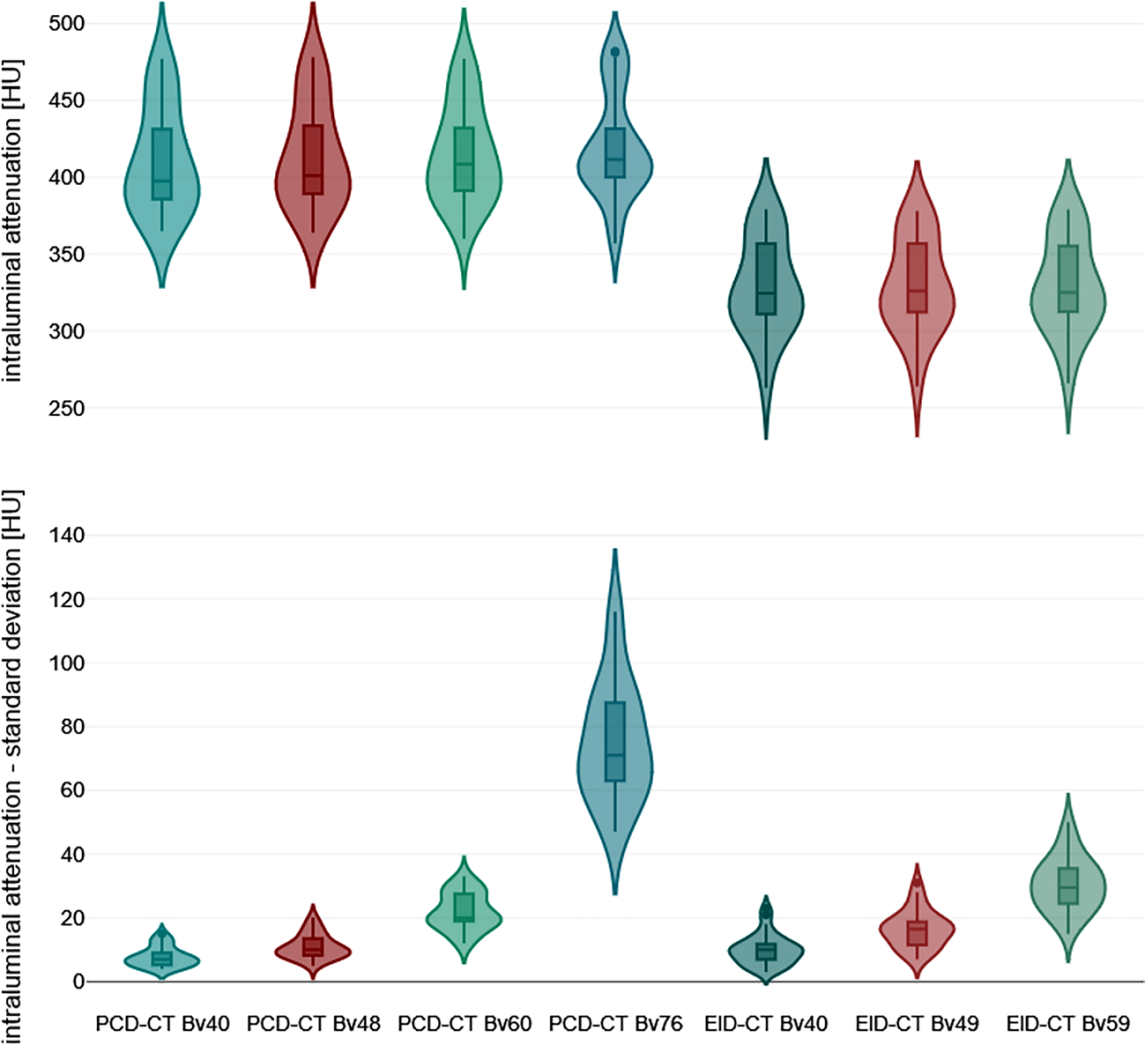
Violine chart illustrating intraluminal attenuation and image noise differences between photon-counting and energy-integrating detector CT.

**Figure 4:**
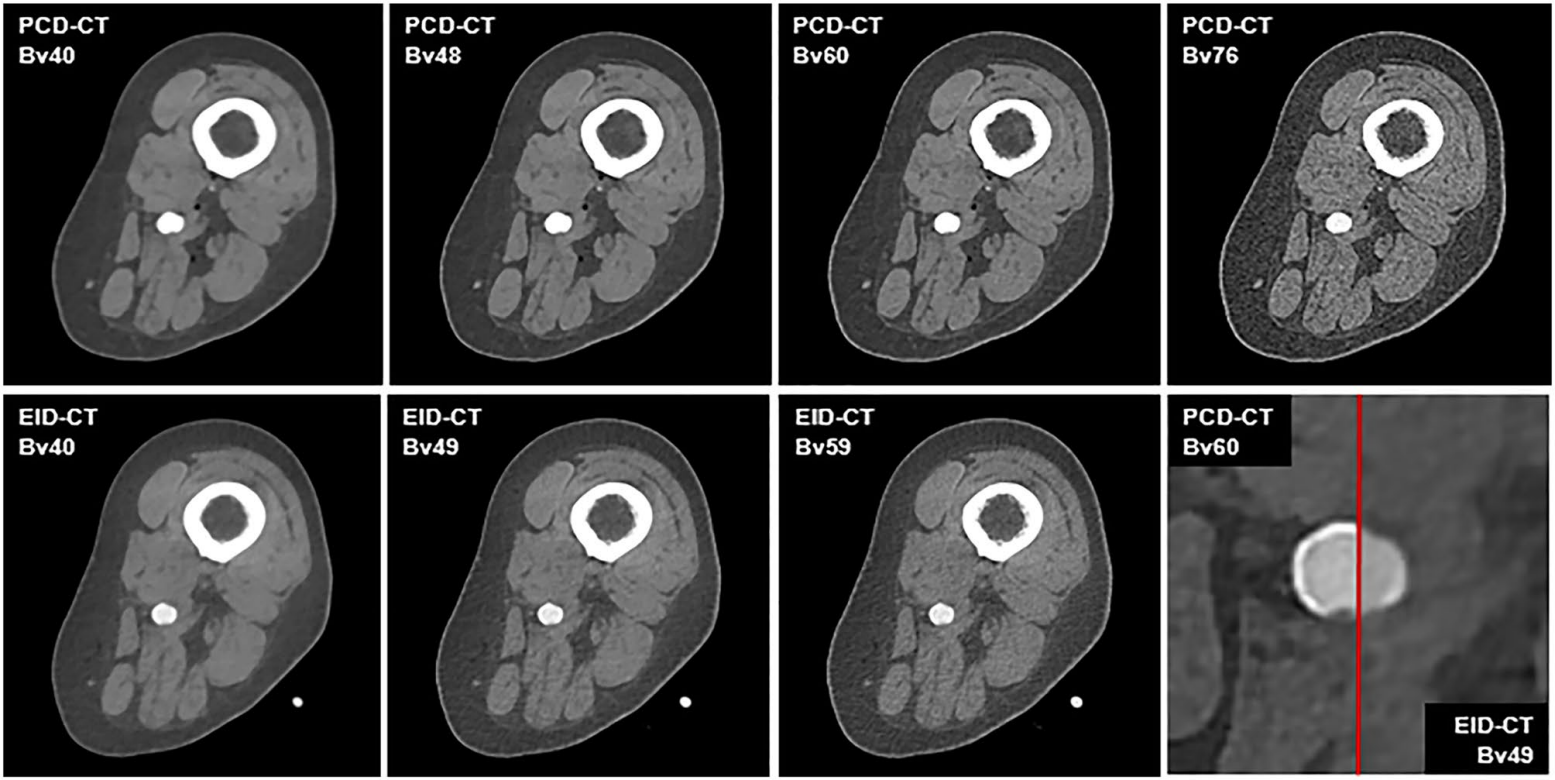
Image quality comparison between photon-counting and energy-integrating-detector CT. *Upper row* Photon-counting detector CT (PCD-CT). *Lower row* Energy-integrating detector CT (EID-CT). *Lower right corner* Direct comparison of a sharp kernel PCD-CT image (Bv60; left half) and a medium-sharp kernel EID-CT image (Bv49; right half) with equivalent SNR/CNR values in the same individual.

The intraluminal attenuation showed no significant differences within the PCD-CT (p ≥ 0.100) or EID-CT group (p ≥ 0.591) for different kernels, while PCD-CT exceeded EID-CT attenuation (414.7 ± 27.3 HU vs. 329.3 ± 24.5 HU; p < 0.001). Image noise (intraluminal) increased significantly with sharper kernels for both scanners, regardless of measurement location (PCD-CT Bv40/48/60: 7.7 ± 3.1/11.0 ± 3.7/22.0 ± 5.7 HU vs. EID-CT Bv40/49/59: 10.0 ± 4.9/16.5 ± 5.7/30.5 ± 8.3 HU; p < 0.001). However, PCD-CT provided significantly lower arterial noise levels compared to EID-CT for matched kernels (p ≤ 0.044) as is exemplified in Fig. 3. The increase in noise to the next sharper kernel was also significantly lower in PCD-CT than in EID-CT. Representative CT images of one body donor reconstructed with all investigated convolution kernels are given in Fig. 4.

**Figure 5:**
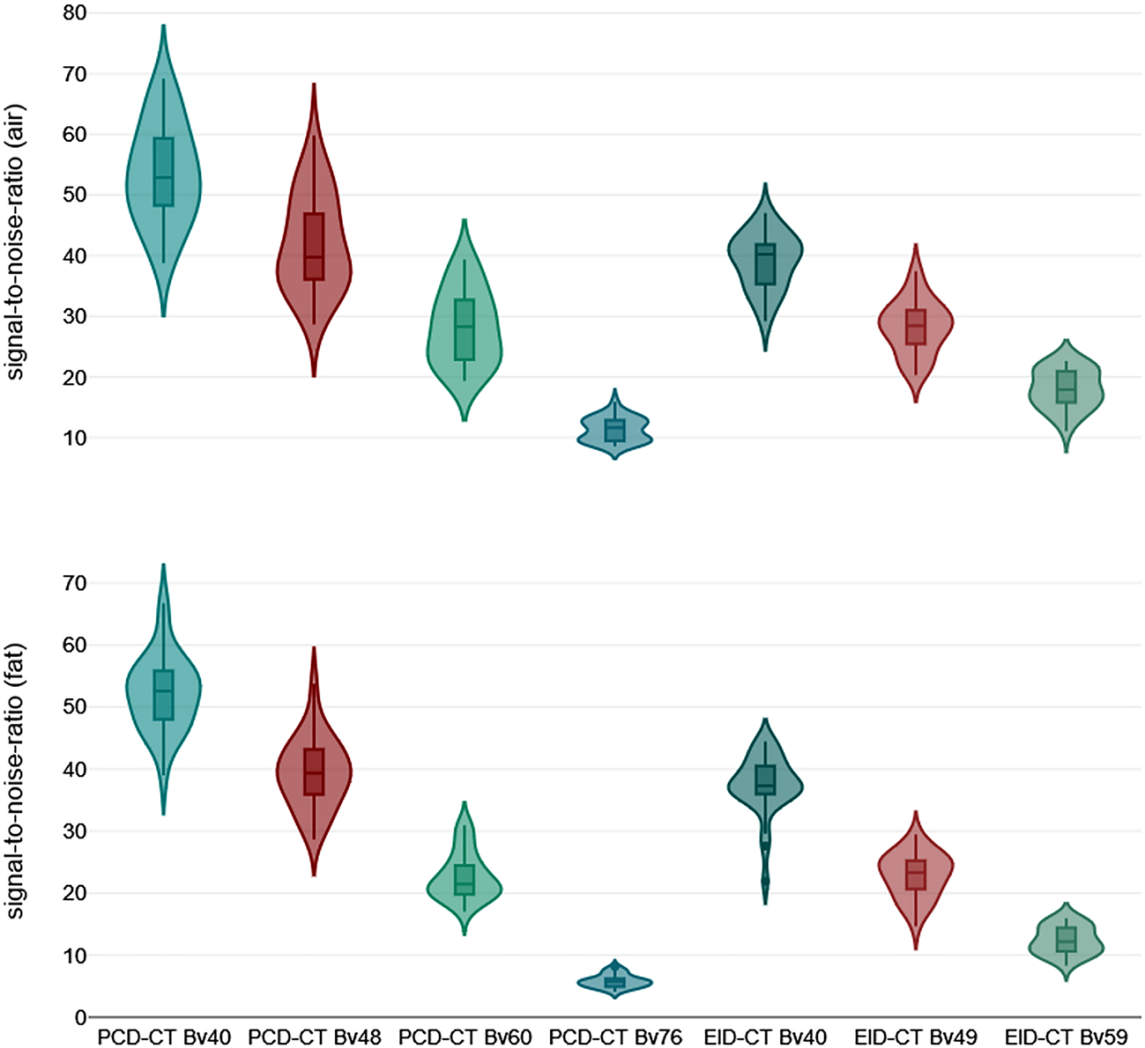
Violine chart illustrating signal-to-noise differences between photon-counting and energy-integrating detector CT.

**Figure 6:**
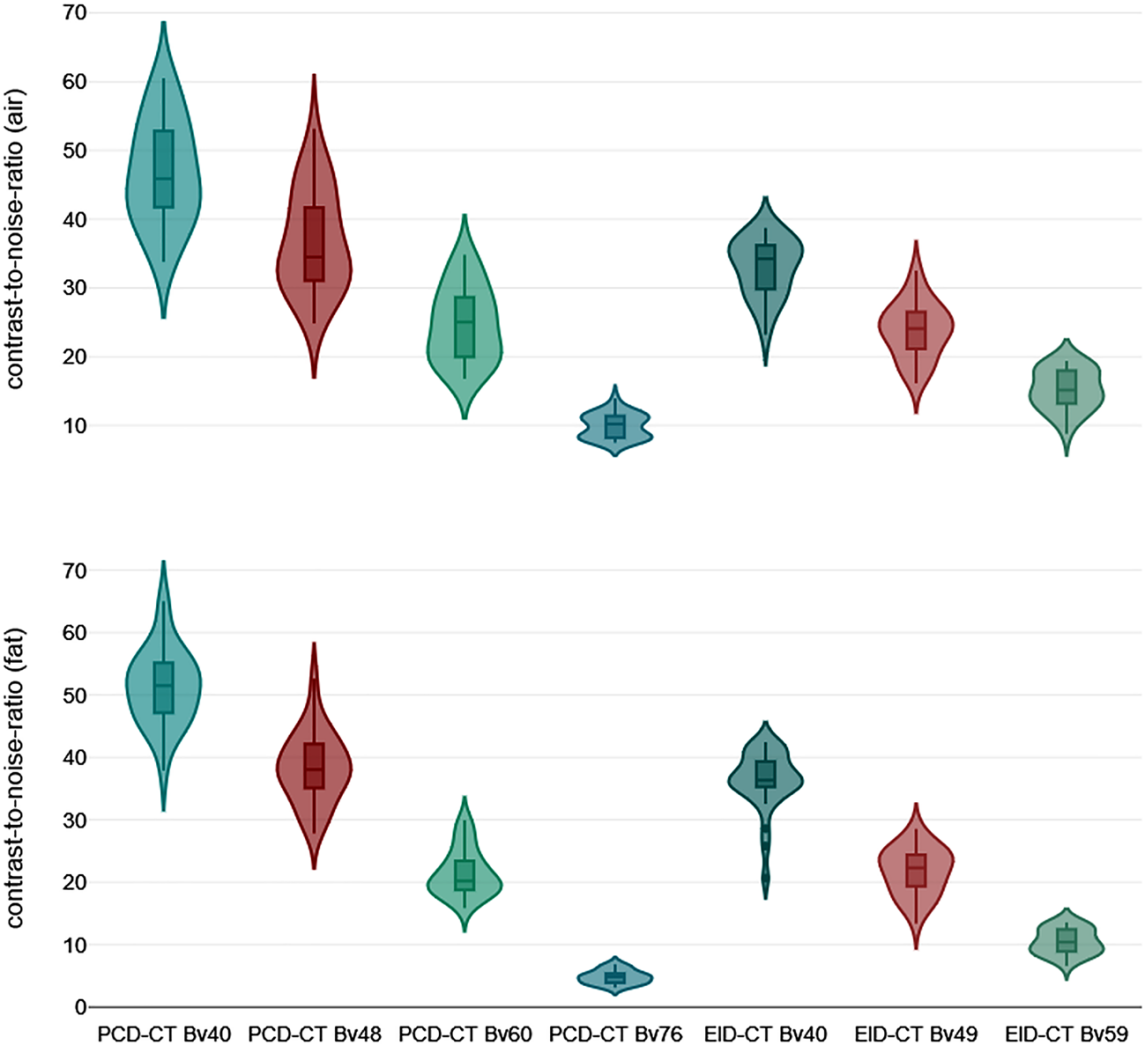
Violine chart illustrating contrast-to-noise differences between photon-counting and energy-integrating detector CT.

SNR_*fat*_ (PCD-CT—Bv40/48/60: 22.7 ± 3.0/39.3 ± 5.5/52.3 ± 4.6 versus EID-CT—Bv40/49/59: 12.4 ± 1.9/22.8 ± 3.5/37.4 ± 3.3) and CNR_*fat*_ (21.5 ± 3.0/38.2 ± 5.4/51.3 ± 4.6 versus 10.5 ± 1.7/21.7 ± 3.7/36.3 ± 3.3) were significantly higher in PCD-CT than in the EID-CT at comparable kernels (p < 0.001). Of note, the next sharper reconstruction kernel with PCD-CT reached the approximate values of the lower EID-CT kernel (e.g. SNR_*fat*_; EID-CT – Bv49: 22.8 ± 3.5 versus PCD-CT – Bv60: 21.4 ± 3.5) (Figs. 5 and 6). Detailed measurements of attenuation and noise, as well as computations of SNR and CNR are summarized in Table 1.

**Table 1:** Summary of the objective quality assessment.

Kernel	Att_lum_ [HU]	Image noise [HU]	SNR_fat_	CNR_fat_	SNR_air_	CNR_air_	SD_fat_ [HU]	SD_air_ [HU]
PCD-CT								
Bv40	410.9 ± 33.9	7.7 ± 3.1	52.3 ± 5.8	51.3 ± 5.9	53.5 ± 8.0	46.7 ± 7.4	7.9 ± 0.9	7.8 ± 0.9
Bv48	413.8 ± 33.2	11.0 ± 3.7	39.3 ± 5.5	38.2 ± 5.4	42.0 ± 7.8	36.8 ± 7.2	10.7 ± 1.6	10.1 ± 1.3
Bv60	414.5 ± 31.1	22.0 ± 5.7	22.7 ± 3.7	21.5 ± 3.7	28.1 ± 5.9	24.6 ± 5.3	18.6 ± 2.3	15.3 ± 2.7
Bv76	419.5 ± 33.3	75.0 ± 2.6	5.8 ± 1.0	4.8 ± 1.0	11.3 ± 1.9	9.9 ± 1.7	73.3 ± 9.6	37.5 ± 4.6
EID-CT								
Bv40	328.9 ± 30.2	10.0 ± 4.9	37.4 ± 4.8	36.3 ± 4.7	39.1 ± 4.4	33.0 ± 4.0	8.9 ± 1.1	8.5 ± 0.8
Bv49	329.4 ± 29.5	16.5 ± 5.7	22.8 ± 3.5	21.7 ± 3.7	28.2 ± 4.3	23.8 ± 3.9	14.7 ± 1.8	11.8 ± 1.3
Bv59	329.6 ± 29.5	30.5 ± 8.3	12.4 ± 2.2	10.5 ± 2.0	18.0 ± 3.1	15.2 ± 2.9	27.2 ± 3.6	18.7 ± 2.5

*PCD-CT* photon-counting detector CT, *EID-CT* energy-integrating detector CT, *Att*_*lum*_ intraluminal attenuation, *HU* Hounsfield units, Image Noise corresponds to standard deviation of Att_lum_; *SNR* signal-to-noise ratio, *CNR* contrast-to-noise ratio, *SD* standard deviation.

### Subjective image analysis

**Figure 7:**
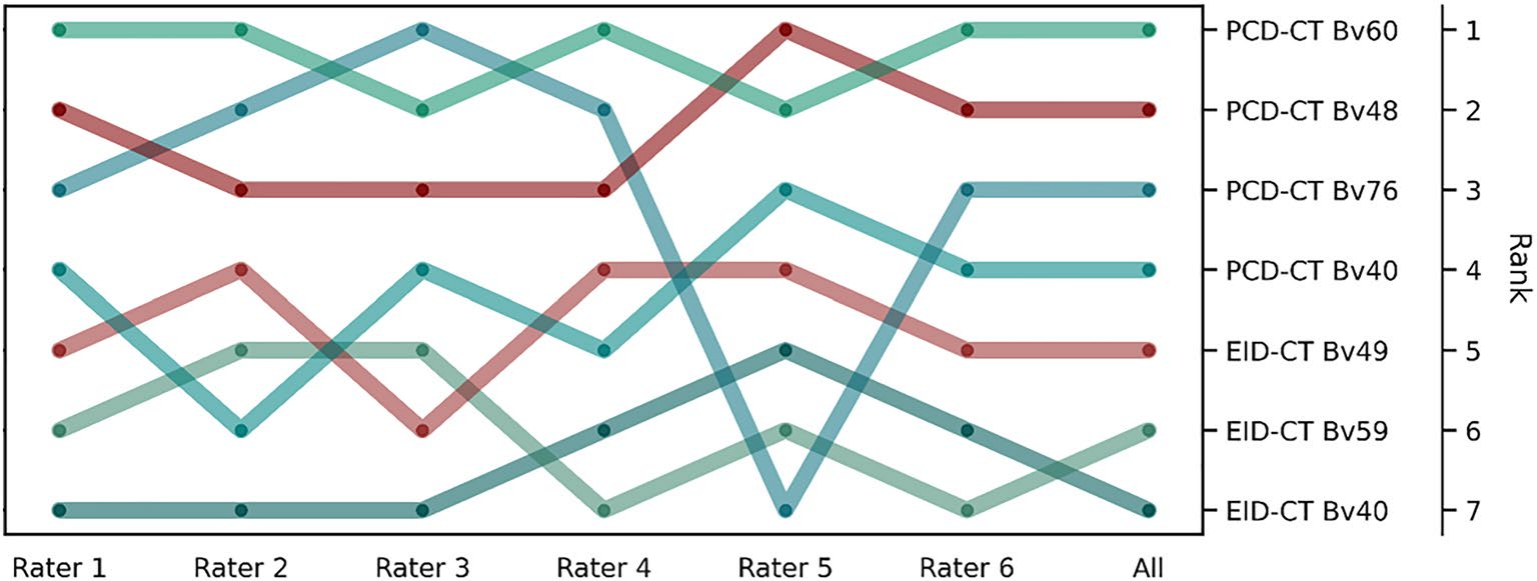
Bump chart of the subjective ratings of the image quality. The bump chart displays the ranks based on the pairwise forced-choice image quality assessment for each scanner/convolution kernel combination by the individual radiologists (Rater 1 to 6) and their averaged rating (All).

Overall image quality of CTA acquired with PCD-CT was favored over EID-CT in rank-wise comparison, irrespective of convolution kernels. With the sole exception of one rater, the PCD-CT scan with the ultra-sharp, vessel-optimized vascular kernel (Bv76) was rated higher than all available EID-CT datasets (Fig. 7). Image quality of PCD-CT scans reformatted with Bv60 received the highest ratings, while EID-CT scans with Bv40 were rated worst. The reader agreement was good (*W* = 0.78) and the differences found were significant (p < 0.001).

## Discussion

Establishing continuous extracorporeal perfusion in a human cadaveric model, this investigation compared kernel-related image quality of dose-matched 120 kVp CTA of the upper leg runoff between a 1st-generation PCD-CT and 3rd-generation EID-CT system. Our results confirm the superiority of the new detector technology regardless of the used convolution kernel in objective analysis, as well as subjective image ratings using a browser-based pairwise forced-choice comparison setup.

Previous feasibility studies have established the value of cadaveric models, which permit realistic CTA imaging and intra-individual comparative studies^27^. Suchlike setups allow for accurate direct comparisons of different scanner types, as characteristic sources of bias in matched-pair patient studies are absent. Otherwise, relevant confounders include different body weight, varying contrast agent amount and concentration, and variations in applied effective radiation dose^28^. Recent studies investigating CTA of the aorta^19^ or peripheral arterial runoffs^24^ have been based on retrospective data and therefore suffer from discrepancies in acquisition protocols between PCD-CT and EID-CT. To overcome this limitation, this experimental cadaveric study employed a constant tube potential of 120 kVp without automatic tube modulation and a fixed tube current resulting in identical CTDI_Vol_ values.

Regardless of utilized convolution kernels, intraluminal attenuation was higher in PCD-CT than in EID-CT datasets. This result is consistent with previous studies^19,24^ and is most likely due to the equal weighting of all photons regardless of their energy by the photon-counting detector and the resulting relative overweighting of low-energy photons compared to EID-CT^28^. Consistent with the above-mentioned previous studies, image noise was lower for comparable kernels in PCD-CT scans and increased with sharper reconstruction kernels. However, the increase in noise remained significantly lower in PCD-CT than in EID-CT. A plausible explanation to this finding lies in the combination of increased beam yield and threshold-based suppression of low-energy electronic noise inherent to the improved detector technology^9^. We also demonstrated that both SNR and CNR for comparable kernels in PCD-CT were significantly higher than with corresponding EID-CT datasets. Thereby, the values of PCD-CT reformatted with the next sharper kernel were comparable to the lower EID-CT kernel. This allows for the use of sharper kernels without a reduction in image quality, facilitating improved vessel assessment, especially in the presence of calcifications or inserted stents. This improves the evaluability of the arterial runoff while maintaining the assessability of the surrounding soft tissue.

Consistent with the results of the objective image quality analysis, all PCD-CT scans were attributed with better subjective ratings than EID-CT scans. However, the fact that one reader ranked images reconstructed with the PCD-specific ultra-sharp kernel worst needs to be mentioned. While this result may be attributed to individual taste, these images show an increased degree of hardening, and thus supposedly allow for sharper delineation of vessel lumina and stenosis evaluation in the presence of calcifications. However, the assessment of the surrounding tissue is certainly limited. Of note, this convolution kernel was primarily used for comparative reasons and requires further dedicated evaluation to assess suitable clinical applications.

In this context, future research is required to investigate the impact of PCD-CTA’s superior image quality on patient treatment by potentially increasing diagnostic accuracy for vascular pathologies, such as stenoses or occlusions. Considering that the potential benefits of ultra-high-resolution PCD-CT examinations providing spectral image information without dose penalty have been demonstrated in cardiac imaging^12,14,16^, dedicated investigation concerning peripheral arterial run-offs is warranted.

Certain limitations specific to this study must be mentioned. First, due to the experimental nature of this work, only 8 extremities of 4 body donors were investigated. Second, to increase comparability, we refrained from applying automatic tube voltage modulation on either scanner, although this could have resulted in a better iodine contrast in EID-CT, as the automatic modulation would have selected lower tube voltages between 70 and 100 kVp. Since fixed tube voltages below 100 kVp were not applicable with PCD-CT at the time of writing, performing low-kV scans solely on the EID-CT scanner would have prevented reasonable comparisons between the two detector techniques in our opinion. Further studies will be necessary to investigate other scan modes with different tube voltage settings once they become applicable. Third, we did not perform PCD-CT examinations in ultra-high resolution scan mode, since this setting could not be replicated on the EID-CT scanner without a considerable dose increase. Finally, to investigate the isolated influence of different convolution kernels, post-processing techniques like virtual monoenergetic imaging or polyenergetic bin imaging were not assessed, since the necessary reconstruction algorithms are not directly comparable.

## Conclusion

PCD-CT offers superior intraluminal attenuation, SNR, and CNR compared to EID-CT in angiographies of the peripheral arterial runoff of the lower extremity. This allows for employing sharper convolution kernels without compromising image quality, hence improving both subjective image quality and assessability of vascular structures as well as surrounding soft tissue.

## Supplementary Information

Supplementary Table S1.

## Data Availability

The datasets generated and/or analyzed during the current study are not publicly available but are available from the corresponding author on reasonable request. Due to the nature of this research, participants of this study did not agree for their data to be shared in a public repository.

## Abbreviations

CNR: Contrast-to-noise ratio
CTA: Computed tomography angiography
CTDI_Vol_: Volume computed tomography dose index
EID-CT: Energy-integrating detector CT
PCD-CT: Photon-counting detector CT
ROI: Region of interest
SNR: Signal-to-noise ratio
