# Supplementary material for: Standardized assessment of vascular reconstruction kernels in photon-counting CT angiographies of the leg using a continuous extracorporeal perfusion model
Source: Sci Rep. 2023 Jul 26;13:12109. doi: 10.1038/s41598-023-39063-z (PMC10372012; doi:10.1038/s41598-023-39063-z)
Supplement: Supplementary file 1 — Supplementary Table S1. [file 41598_2023_39063_MOESM1_ESM.docx]

**Table S1 – Modulation transfer functions of the employed convolution kernels**

| **Kernel** | | **ρ_50_** *[lp/cm]* | **Δ** *[%]* | **ρ_10_** *[lp/cm]* | **Δ** *[%]* | **ρ_02_** *[lp/cm]* | **Δ** *[%]* |
| --- | --- | --- | --- | --- | --- | --- | --- |
| **PCD-CT** | Bv40 | 3.95 | 0.0 | 6.61 | 0.0 | 8.02 | 0.0 |
|  | Bv48 | 5.40 | 3.9 | 8.49 | 1.2 | 9.88 | 0.3 |
|  | Bv60 | 8.79 | 5.6 | 11.86 | 1.1 | 12.23 | 0.4 |
|  | Bv76 | 16.47 | --- | 22.12 | --- | 24.93 | --- |
| **EID-CT** | Bv40 | 3.95 | 0.0 | 6.61 | 0.0 | 8.02 | 0.0 |
|  | Bv49 | 5.62 | 4.0 | 8.59 | 1.2 | 9.91 | 0.3 |
|  | Bv59 | 8.32 | 5.3 | 11.73 | 1.1 | 12.18 | 0.4 |

**PCD-CT** photon-counting-detector CT; **EID-CT** energy-integrating-detector CT; **ρ_50/10/02_** spatial frequency (1/cm) at the 50% / 10% / 2% value of the modulation transfer function (MTF) in line pairs per cm (**lp/cm**); **Δ** difference to the compared kernel in percent
